# From One of Our Co-Laborers

**Published:** 1873-05

**Authors:** 


*From one of our Co-Laborers.*

"We agree with you in the opinion that an advertising department in a medical journal is a necessity, in order to fully meet the wants of the profession. Without pure medicine, it matters but little what we prescribe. A journal teaching us the character of diseases, the science and art of prescribing for their cure, would be unnecessary; hence, it is the duty of physicians to feel an interest in their home druggist. They should learn from reliable journals and other means who manufacture and prepare the purest and most reliable drugs and medicines, and advise their local druggist to purchase alone from parties who are known to be reliable. This much is due to themselves, to the profession, and to the sick. Cheap and impure drugs are often the cause of the loss of life and reputation. Without good books, suitable instruments, and pure drugs, as a profession we can accomplish but little. Your advertisements for artificial limbs, we are satisfied, have done much good."

The views above have been frequently expressed by us, editorially; and we still think the importance and value of a well-arranged advertising department in a medical journal is of incalculable advantage to practitioners of medicine. It is our desire to monthly lay before them the very best medical truths found in the current medical literature of the day, and while every effort shall be made in this direction, we, at the same time, deem it no less a duty to point them to first-class, reliable houses from which to draw their supplies—knowing full well that no treasury of medical truths, no powers of diagnosis and correct principles of treatment of diseases, will avail the practitioner in his hour of need, if his chemicals are impure and his drugs worthless.

This being our wish, we would be pleased to have any of our friends and co-laborers suggest the names of houses they would desire to see advertised in this journal—houses they know to be reliable and worthy of the patronage of our large circle of readers. This much we ask for the sake of all—both readers, advertisers and journal—that, being bound together in mutual interests, the interests of all may be subserved and promoted, honestly and faithfully. We have already furnished our friends the names of some first-class business houses, to which we now specially desire to call their attention:

*Tilden & Co.*—This old-established house has earned a reputation for reliability second to no other in this country—a fact we have frequently done them the justice to mention.

*Alexander Brown & Co., Manufacturers of Cold Expressed Fluid Extracts, Elixirs, etc.,* 124 *Vine Street, Philadelphia, Penn.*—We specially ask the attention of those of our friends desiring reliable drugs and preparations to this firm. This house will be found prompt and reliable. Their fluid extracts are put up and priced (see advertisement) in a form most convenient for the practitioner, who may order them direct by forwarding the prices annexed. Look over the large list, and see if you do not need something they offer.

*Codman & Shurtleff.*—This old and established house is well-known in every State and Territory of the Union. As manufacturers of the Steam Atomizer and other apparatus of the kind, as well as instruments indispensable to the surgeon and physician, they have acquired a reputation honorable and reliable in the highest degree. Should our friends need—and they certainly do—anything in their line, send them your orders. You will be honorably dealt with and promptly attended to. Their atomizing apparatus are for sale at the well-known drug-store of Messrs. Redwine & Fox, Atlanta, Georgia.

*Galvano-Faradic Manufacturing Company,* 167 *East Thirty-fourth Street, New York.*—The importance of the surgical and therapeutical application of electricity in the treatment of diseases can not be well overstated. Every practitioner should possess his "battery." The time is near at hand when to be without the means of employing electricity will detract from the reputation of the surgeon and physician. If you want good machines—portable and serviceable—remember the Galvano-Faradic Manufacturing Company will be *sure* to furnish them.

*J. H. Gemrig, Manufacturer of Surgical and Orthopædical Instruments, etc.,* 109 *South Eighth Street, Philadelphia.*—This gentleman is a skillful and reliable instrument-maker, who will take pleasure in making and forwarding any instrument or instruments desired. Give him a trial, and you will be pleased.

*Samuel S. White, Chestnut Street, Corner Twelfth, Philadelphia.*—

This house is devoted to specialties for physicians, and practitioners everywhere would do well to read the advertisement. Orders will be attended to promptly and "on the square."

*Henry K. Wampole & Co., Wholesale Druggists,* 123 *North Fourth Street, above Arch, Philadelphia.*—Call the attention of your druggist to this house. You will get pure drugs should you order from them.

*A. M. Leslie & Co.,* 319 *North Fifth Street, St. Louis, Mo.*—Our Western and Southwestern readers will do well to remember Leslie & Co. when about to order their supplies.

*Dr. Clements* and *H. A. Gildea,* both of Philadelphia, offer fine inducements to the "limbless." Call the attention of the unfortunates who need limbs to these houses—they cannot be better fitted and supplied.

*James W. Queen* and *Joseph Zentmayer* will fit you up microscopes, etc., to your satisfaction in prices and qualities.

*Harlem Lodge*—the inebriate asylum—will, when moral suasion and temperance societies fail, give your patients a chance for a return to decency and self-respect. "The love of strong drink" is a disease, to be met and subdued by well-directed measures, which can be carried into prompt execution at Harlem Lodge.

*Home Advertisers.*—These have been selected by us as among the best and most reliable business houses of Atlanta. Look over the list: not a name among them all but we can indorse and recommend. If you purchase in this market, give them a trial. You will be pleased.

Owing to want of space, we are enabled to make only special mention of one of our home advertisers in this number—we refer to the excellent and highly valuable steam dyeing and cleaning establishment of James Lochrey, which is one of the most useful and important enterprizes of the "Gate City." Owing to the scarcity of such establishments in the South, many of our friends will confer lasting benefit on their friends and acquaintances by calling their attention to his enterprize. If it be true that economy is the highway to wealth, it becomes their duty to point out the way to those who know it not. Please remember this, and spread the news for the benefit of all parties.

